# Prediction model of in‐hospital cardiac arrest using machine learning in the early phase of hospitalization

**DOI:** 10.1002/kjm2.12895

**Published:** 2024-09-25

**Authors:** Wei‐Tsung Wu, Chew‐Teng Kor, Ming‐Chung Chou, Hui‐Min Hsieh, Wan‐Chih Huang, Wei‐Ling Huang, Shu‐Yen Lin, Ming‐Ru Chen, Tsung‐Hsien Lin

**Affiliations:** ^1^ Division of Cardiology, Department of Internal Medicine Kaohsiung Medical University Hospital Kaohsiung Taiwan; ^2^ Graduate Institute of Medicine, College of Medicine Kaohsiung Medical University Kaohsiung Taiwan; ^3^ Big Data Center Changhua Christian Hospital Changhua City Taiwan; ^4^ Graduate Institute of Clinical Medicine National Chung Hsing University Taichung Taiwan; ^5^ Graduate Institute of Statistics and Information Science National Changhua University of Education Changhua City Taiwan; ^6^ Center for Big Data Research Kaohsiung Medical University Kaohsiung Taiwan; ^7^ Department of Medical Imaging and Radiological Sciences Kaohsiung Medical University Kaohsiung Taiwan; ^8^ Department of Public Health Kaohsiung Medical University Kaohsiung Taiwan; ^9^ Center for quality management and patient safety Kaohsiung Medical University Hospital Kaohsiung Taiwan; ^10^ Department of Internal Medicine, School of Medicine Kaohsiung Medical University Kaohsiung Taiwan

**Keywords:** cardiopulmonary resuscitation, cardioversion, electrical defibrillation, in‐hospital cardiac arrest, machine learning

## Abstract

In hospitals, the deterioration of a patient's condition leading to death is often preceded by physiological abnormalities in the hours to days beforehand. Several risk‐scoring systems have been developed to identify patients at risk of major adverse events; however, such systems often exhibit low sensitivity and specificity. To identify the risk factors associated with in‐hospital cardiac arrest (IHCA), we conducted a retrospective cohort study at a tertiary medical center in Taiwan. Four machine learning algorithms were employed to identify the factors most predictive of IHCA. The support vector machine model was discovered to be the most effective at predicting IHCA. The ten most critical physiological parameters at 8 h prior to the event were pulse rate, age, white blood cell count, lymphocyte count, body temperature, body mass index, systolic and diastolic blood pressure, platelet count, and use of central nervous system‐active medication. Using these parameters, we can enhance early warning and rapid response systems in our hospital, potentially reducing the incidence of IHCA in clinical practice.

## 1 INTRODUCTION

Approximately 3.7% of patients in hospitals experience a severe adverse event, such as a cardiopulmonary arrest, unplanned admission to the intensive care unit (ICU), or sudden death.^1^ In‐hospital cardiac arrest (IHCA) is an acute event that can affect any hospitalized patient. IHCA is generally associated with significant morbidity and mortality. Despite medical efforts, the overall 1‐year survival rate in patients with IHCA remains relatively low, with only 13% of patients surviving to be discharged.^2^ Unlike out‐of‐hospital cardiac arrest (OHCA), IHCA can usually be predicted and prevented because hospitalized patients often have multiple comorbidities, and the clinical conditions of hospitalized patients are continually being monitored. Studies and retrospective reviews have indicated that abnormal performance, vital signs, and biochemical markers are commonly abnormal before an IHCA.^3^, ^4^, ^5^ Several laboratory parameters that independently predict the risk of poor health outcomes and cardiac arrest have been identified.^6^ Several factors, including patient variables (i.e., age, sex, and comorbidities) and cardiac arrest characteristics (i.e., initial rhythm, duration of resuscitation, and event location) affect IHCA prognosis.^7^ Several models have been developed for predicting IHCA prognos is by using these factors.^8^, ^9^ The findings of these studies have led to the widespread adoption of rapid response teams, the aim of which is to prevent cardiac arrest on hospital wards through early intervention and optimal treatment.

The use of machine learning (ML) for developing prediction models has gained considerable attention recently due to the ability of ML to learn complex relationships between multiple variables without a reliance on assumptions such as independence and linearity. Scholars have constructed ML‐based models for predicting cardiac arrest by using electrocardiogram (ECG) recordings and ECG‐based heart rate variability measures.^10^, ^11^ Laboratory data are objective and easily accessible information. One study employed only laboratory parameters to develop an ML‐based model for predicting IHCA in rescue‐treated patients, with its findings demonstrating the reliability of this approach.^4^ Another study integrated routine laboratory data with vital signs to construct an ML model for predicting IHCA in hospitalized adult patients for more than 24 h; however, the use of laboratory data plus vital signs did not result in significantly higher prediction performance than did the use of vital signs only.^12^ These studies have highlighted the potential of ML algorithms constructed using routine laboratory data and vital signs for predicting IHCA. However, use of medications, such as cardiovascular and central nervous system (CNS)‐active medications, is also potentially important information for predicting IHCA, and medication details have not been previously integrated into ML‐based models for predicting IHCA. Moreover, ML research focusing on predicting IHCA by integrating routine laboratory data, vital signs, and medication parameters has been relatively scarce.

In this study, we aim to develop and validate artificial intelligence (AI) algorithms based on ML techniques for predicting IHCA in patients who received cardiopulmonary resuscitation. The predictive factors explored by the model were laboratory data, vital signs, and medication use.

## 2 MATERIALS AND METHODS

### 2.1 Study design and data source

We conducted a retrospective cohort study at Kaohsiung Medical University Hospital (KMUH), a tertiary medical center in Taiwan. The study population comprised patients who were admitted to the hospital from January 2017 to December 2021. Total enrolled patients were divided into IHCA and non‐IHCA groups. IHCA group was defined as any case involving a hospitalized patient receiving cardiopulmonary resuscitation, electrical defibrillation, or cardioversion. Patients were excluded if they were aged younger than 20 years, died before the discovery of IHCA, or had more than 60% missing data. Missing values for enrolled patients were imputed using patients' median value. We obtained data from KMUH electronic health records. We collected patient demographic data, vital sign data, and laboratory data upon patient admission. The timing for the data collection of the events is 8 h before IHCA for IHCA group and 8 h before discharge for non‐IHCA group. Data on the enrollees' medications, including cardiovascular drugs and CNS‐active medications, were retrospectively collected from electronic medical records. The types of medications used are presented in the Appendix in Table A1. The study protocol was evaluated and approved by the Institutional Review Board of KMUH [KMUHIRB‐E(II)‐20220289].

### 2.2 Machine learning

To construct a model for predicting IHCA, four ML algorithms were employed and compared in this study: support vector machine (SVM), least absolute shrinkage and selection operator (LASSO), ensemble learning with bootstrap aggregation (BAG), and random forest (RF). Training (85%) and testing (15%) datasets were generated using random selection such that the clinical features of patients in the two subsets were matched. Because the numbers of patients with and without IHCA were quite different, the synthetic minority oversampling technique (SMOTE) was performed to balance the IHCA events for the training set. A 10‐fold cross‐validation was performed to construct the prediction model by using the training set, and the model's performance was assessed using the testing set by determining the area under the receiver operating characteristic (ROC) curve. Moreover, the leave‐one‐out approach was employed to search for the 10 features that most strongly predicted IHCA occurrence. The software used to perform ML was Spyder IDE version 5.3.3 (Spyder, Scientific Python Development Environment).

### 2.3 Statistical analysis

We used descriptive statistics to analyze the study population and IHCA status. Data are reported as means and standard deviations for continuous variables and as percentages for categorical variables. For comparisons of groups with and without IHCA, the *χ* ^2^ test was used for categorical variables, and an independent *t* test was used for continuous variables (Table 1). In all tests, *p* < 0.05 was considered statistically significant. Statistical analys is were performed using SAS version 9.4 (SAS Institute, Cary, NC, USA).

**TABLE 1:** Baseline characteristics of patients with and without IHCA.

	Non‐IHCA	IHCA	*p*‐valve
Demographic (mean ± SD)			
Age	55.5 ± 17.4	63.5 ± 16.3	<0.0001
Gender = male	15,609 (48.09%)	155 (64.58%)	<0.0001
BMI, kg/m^2^	24.8 ± 4.4	24.4 ± 4.8	0.2644
Days of stay	6.0 ± 6.0	14.0 ± 18.4	<0.0001
Vital signs			
Vital signs at admission (mean ± SD)			
SBP, mmHg	135.3 ± 29.7	131.1 ± 26.0	0.0129
DBP, mmHg	80.8 ± 12.8	77.8 ± 16.1	0.0047
Pulse rate, beats per minute	79.3 ± 18.3	85.9 ± 25.4	<0.0001
Respiratory rate, breaths per minute	17.5 ± 2.5	18.2 ± 3.9	0.0031
Body temperature, Celsius	36.7 ± 1.1	36.4 ± 1.3	0.0005
SpO_2_, %	97.4 ± 3.3	96.7 ± 4.2	0.0255
Vital signs before 8 h of the event (mean ± SD)			
SBP, mmHg	128.7 ± 19.2	135.4 ± 56.9	0.0675
DBP, mmHg	77.3 ± 12.3	78.7 ± 17.2	0.2191
Pulse rate, beats per minute	73.3 ± 13.9	85.1 ± 24.5	<0.0001
Respiratory rate, breaths per minute	17.6 ± 1.9	18.2 ± 3.8	0.016
Body temperature, Celsius	36.5 ± 0.7	36.2 ± 2.7	0.1094
SpO_2_, %	97.0 ± 7.2	96.6 ± 4.1	0.1496
Laboratory results			
Laboratory results at admission (mean ± SD)			
Potassium, mmol/L	3.8 ± 0.8	4.0 ± 1.3	0.0747
Sodium, mmol/L	136.8 ± 4.4	136.8 ± 6.2	0.9796
Creatinine, mg/ dL	1.1 ± 1.2	1.7 ± 1.6	<0.0001
BUN, mg/dL	16.8 ± 13.1	24.0 ± 18.9	<0.0001
C‐reactive protein, mg/dL	28.9 ± 49.0	48.5 ± 87.0	0.0006
Hemoglobin, g/dL	13.1 ± 1.9	12.5 ± 2.7	0.0005
Red blood cell, 10^6^/uL	4.5 ± 0.6	4.2 ± 0.9	0.0002
White blood cell, 103/uL	9.5 ± 5.4	14.0 ± 27.3	0.0129
HCT, %	39.1 ± 5.2	37.8 ± 7.9	0.0181
MCV, fL	88.4 ± 6.5	90.4 ± 9.1	0.0008
MCH, pg/cell	29.6 ± 2.7	29.8 ± 3.4	0.5287
MCHC, g/dL	33.4 ± 1.2	32.9 ± 1.8	<0.0001
RDW, %	13.3 ± 1.2	13.9 ± 2.1	<0.0001
Platelet, 10^9^/L	243.8 ± 70.0	222.2 ± 92.6	0.0004
WBC classification			
Lymphocyte, %	17.6 ± 7.3	21.1 ± 15.8	0.0007
Monocyte, %	5.5 ± 1.7	5.2 ± 2.0	0.0505
Eosinophil, %	1.0 ± 1.4	1.0 ± 1.5	0.645
Basophil, %	0.3 ± 0.2	0.3 ± 0.3	0.0911
Laboratory results before 8 h of the event, mean ± SD			
Potassium, mmol/L	3.9 ± 0.8	4.0 ± 1.3	0.1241
Sodium, mmol/L	137.3 ± 4.8	136.8 ± 6.4	0.2365
Creatinine, mg/dL	1.1 ± 1.3	1.7 ± 1.6	<0.0001
BUN, mg/dL	16.7 ± 14.1	24.5 ± 19.4	<0.0001
C‐reactive protein, mg/dL	25.3 ± 51.4	46.7 ± 88.2	0.0002
Hemoglobin, g/dL	13.2 ± 2.6	12.5 ± 2.8	<0.0001
Red blood cell, 10^6^/uL	4.5 ± 0.8	4.2 ± 1.0	<0.0001
White blood cell, 103/uL	9.0 ± 5.8	13.9 ± 27.4	0.0053
HCT, %	39.6 ± 6.1	37.8 ± 8.1	0.0012
MCV, fL	88.0 ± 8.1	90.5 ± 9.3	<0.0001
MCH, pg/cell	29.4 ± 3.4	29.8 ± 3.5	0.0314
MCHC, g/dL	33.3 ± 1.5	32.9 ± 1.8	0.0012
RDW, %	13.4 ± 1.4	13.9 ± 2.2	0.0002
Platelet, 10^9^/L	252.3 ± 84.5	223.1 ± 96.9	<0.0001
WBC classification			
Lymphocyte, %	21.1 ± 9.1	22.4 ± 15.9	0.2301
Monocyte, %	5.6 ± 1.9	5.4 ± 2.2	0.068
Eosinophil, %	1.4 ± 1.8	1.2 ± 1.7	0.0912
Basophil, %	0.4 ± 0.3	0.3 ± 0.4	0.0003
Medication use			
Cardiovascular medications	13,430 (41.38%)	183 (76.25%)	<0.0001
CNS‐active medications	16,666 (51.34%)	156 (65.00%)	<0.0001
Others	20,167 (62.13%)	188 (78.33%)	<0.0001

Abbreviations: 8‐h of the event, 8 h before IHCA for IHCA group or discharge in non‐IHCA group; BMI, body mass index; BUN, blood urea nitrogen; CNS, central nervous system; DBP, diastolic blood pressure; HCT, hematocrit; IHCA, in‐hospital cardiac arrest; MCH, mean corpuscular hemoglobin; MCHC, mean corpuscular hemoglobin concentration; MCV, mean corpuscular volume; RDW, red blood cell distribution width; SBP, systolic blood pressure.

## 3 RESULTS

A total of 32,719 hospitalized patients were enrolled (32,459 patients without IHCA and 260 patients with IHCA). A total of 20 patients with IHCA were excluded due to the exclusion criteria. Patients with IHCA were more likely to be male (64.58% vs. 48.09%, *p* < 0.0001), were older (63.5 vs. 55.5 years, *p* < 0.0001), and were longer admission (14 vs. 6 days, *p* < 0.0001) than patients without IHCA. Systolic and diastolic blood pressure upon admission was much lower in the IHCA group than in the non‐IHCA group (131/77.8 mmHg vs. 135.3/80.8 mmHg, *p* < 0.001). SpO_2_ level was significantly higher (97.4% vs. 96.7%, *p* = 0.0255) in the non‐IHCA group than in the IHCA group. Pulse rate upon admission and before 8 h of the event was significantly higher in the IHCA group (85.9 bpm vs. 79.3 bpm, *p* < 0.0001 and 85.1 bpm vs. 73.3 bpm, *p* < 0.0001, respectively); however, systolic and diastolic blood pressure was not different between the groups. A comparison of basic patient characteristics, including vital signs and laboratory results upon admission and 8 h before the event, is presented in Table 1.

**FIGURE 1:**
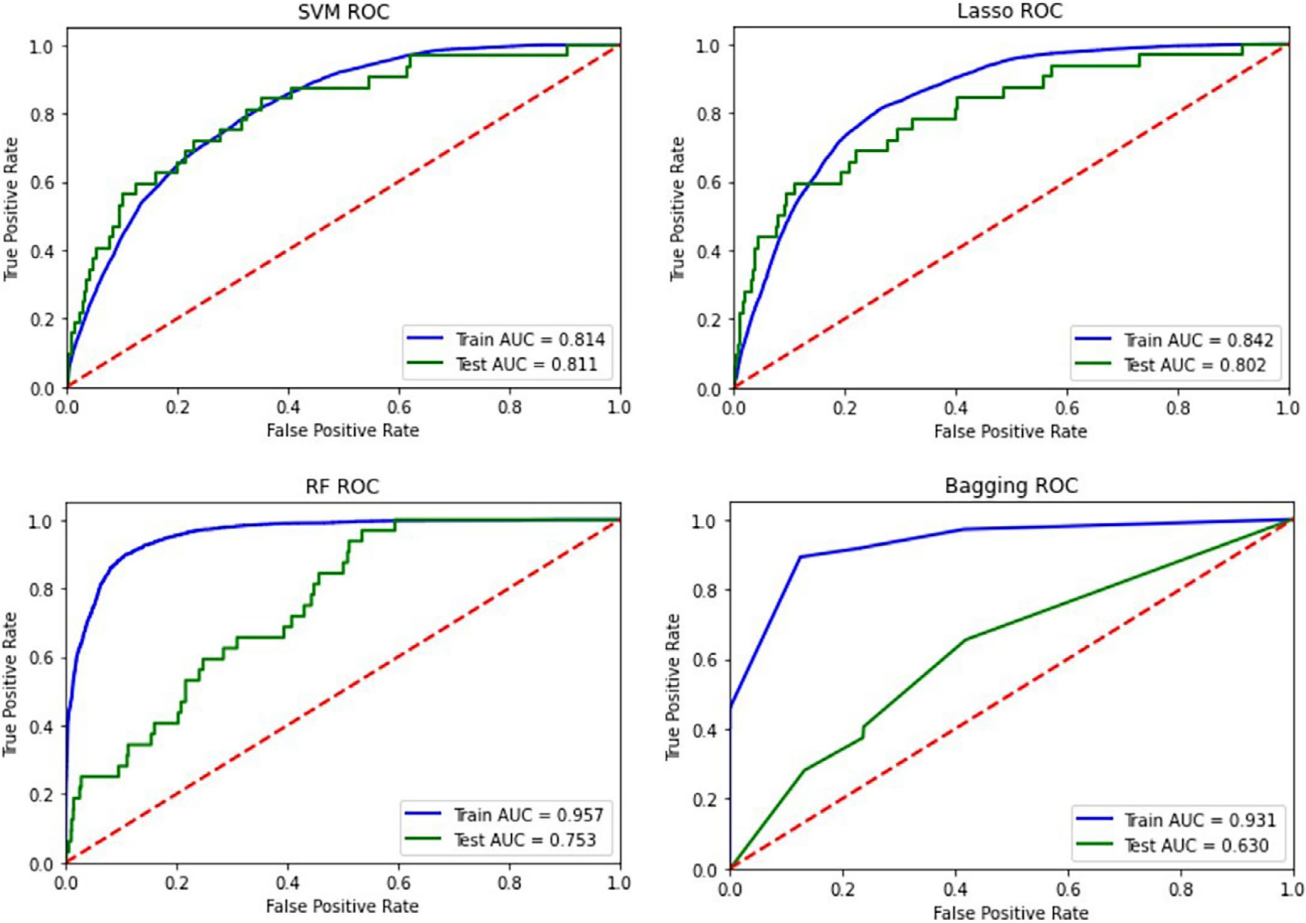
ROC Curve of Four Machine Learning Algorithms for Predicting IHCA in Training and Testing Sets. AUC, area under the ROC curve; Bagging, ensemble learning with bootstrap aggregation; LASSO, least absolute shrinkage and selection operator; RF, random forest; ROC, receiver operating characteristic; SVM, support vector machine.

The training set comprised 27,794 patients (non‐IHCA:IHCA ratio = 27,590:204), and the testing set comprised 4905 patients (non‐IHCA:IHCA ratio = 4869:36). The patients in the 2 sets were randomly selected at a ratio of 85%:15%. The SVM, LASSO, BAG, and RF algorithms were employed in models for predicting IHCA. The comparison results and ROC curves for the four ML prediction models are presented in Figure 1. For the training data, the RF algorithm exhibited the highest performance (AUC: 0.957 for RF, 0.931 for BAG, 0.842 for LASSO, and 0.814 for SVM). For the testing data, the SVM algorithm with Gaussian kernel was the best predictor of IHCA (AUC: 0.811 for SVM, 0.802 for LASSO, 0.753 for RF, and 0.630 for BAG; Table 2). Overall, the SVM model exhibited the highest performance for predicting IHCA in the training and testing sets.

**TABLE 2:** Comparison of four ML IHCA prediction algorithms.

	Training set	Testing set
SVM	0.814	0.811
LASSO	0.841	0.802
RF	0.957	0.753
BAG	0.931	0.630

Abbreviations: BAG, ensemble learning with bootstrap aggregation; IHCA, in‐hospital cardiac arrest; LASSO, least absolute shrinkage and selection operator; RF, random forest; SVM, support vector machine.

A feature importance test revealed that the 10 most important features were physiological parameters recorded 8 h prior to the event: pulse rate, age, white blood cell count (WBC), lymphocyte count, body temperature, body mass index (BMI), systolic and diastolic blood pressure, platelet (PLT) count, and CNS‐active medication. The details are displayed in Table 3.

**TABLE 3:** Ten most important features of SVM model for predicting IHCA.

Rank	SVM model of IHCA prediction
1	Pulse rate 8 h
2	Age
3	WBC 8 h
4	Lymphocyte 8 h
5	Body temperature 8 h
6	BMI
7	DBP 8 h
8	PLT 8 h
9	SBP 8 h
10	CNS‐active medications

Abbreviations: 8 h, 8 h before the event; BMI, body mass index; CNS, central nervous system; DBP, diastolic blood pressure; IHCA, in‐hospital cardiac arrest; PLT, platelet count; SBP, systolic blood pressure; SVM, support vector machine; WBC, white blood cell count.

## 4 DISCUSSION

Studies have shown that abnormal vital signs are associated with progression to cardiac arrest.^13^, ^14^ Some abnormal vital signs have also been identified in the hours prior to IHCA.^15^, ^16^ In our study, we used four ML algorithms to identify factors—demographic, vital signs, and laboratory data—that could predict IHCA in KMUH. Through 10‐fold cross‐validation, the SVM algorithm was found to be the best model for predicting IHCA.

Ten physiological parameters were identified as being important predictors. When measured 8 h prior to the event, these parameters were especially important. Pulse rate, age, WBC, lymphocyte count, body temperature, BMI, systolic and diastolic blood pressure, PLT count, and CNS‐active medication were crucial predictors of IHCA.

Several early warning systems (EWSs) are available for detecting clinical worsening of patient conditions^17^; however, early warning systems often have low sensitivity and specificity.^18^ A consensus has not yet been reached on the optimal early warning system for patients in acute care settings.^19^ KMUH employs the National Early Warning Score (NEWS) early warning system; the NEWS incorporates respiratory rate, oxygen saturation, temperature, systolic blood pressure, heart rate, and alert–voice–pain–unresponsive score. The optimal threshold for the NEWS in clinical settings is uncertain. Studies validating the NEWS did not standardize patient outcomes and clinical workloads.^20^

In our study, more variables are included in the NEWS (i.e., age, WBC, lymphocyte count, PLT count, BMI, and CNS‐active medication) provided valuable information for early intervention in patients and increased the accuracy of predictions in patients with IHCA. To reduce in‐hospital mortality rates, IHCA response protocols have been developed.^21^ Rapid response teams have been introduced to reverse the trend of poor outcomes in patients with IHCA. However, the algorithms used by medical emergency teams and rapid response teams are not universal.^22^ Because vital signs and laboratory data are routinely checked to assess the condition of hospitalized patients, these data are easily obtained from hospitalized patients. These physiological parameters at 8 h prior to the event can enable clinicians to provide personalized medical care and can form part of the early warning system.

The main strength of this study lies in its development of reliable ML models that accurately predicted IHCA solely on the basis of readily available laboratory data, vital signs, and medication usage information. By enabling accurate IHCA prediction, our model has the potential to alleviate the strain on frontline medical care and reduce cardiopulmonary resuscitation rates, thereby improving patient outcomes.

## 5 LIMITATIONS

This study has several limitations. First, this was a retrospective study that enrolled patients from a single medical center. Therefore, the study population was homogenous. Additionally, the sample size was relatively small. Second, we did not consider whether IHCA occurred in a ward, intensive care unit, or other location. Different locations and monitoring statuses could result in different early warning signs. Third, our study included a limited number of variables—demographics, vital signs, laboratory results, and medications. Abnormal electrocardiography data are collected in 67% of individuals before a sudden cardiac death.^23^ Other factors that significantly influence the likelihood of IHCA—such as electrocardiogram analysis results, N‐terminal pro‐brain natriuretic peptide, prothrombin time, and activated partial thromboplastin time—were not included in our study.^24^, ^25^

## 6 CONCLUSION

We developed an IHCA prediction model by using ML algorithms for patients in KMUH. Of the various algorithms tested, SVM yielded optimal results. The major risk features identified are mainly physiological parameters 8 h prior to the event and medication usage information. In the future, we aim to incorporate these important features into an early warning system powered by artificial intelligence to prevent IHCA to the greatest extent possible.

## CONFLICT OF INTEREST STATEMENT

All authors declare no conflict of interest.

## Appendix

**TABLE A1:** Types of medications.

No.	Drugs/group of drugs
Cardiovascular medications	
1	Antihypertensives
2	Diuretics
3	Beta‐blocking agents
4	Calcium channel blockers
5	Renin–angiotensin system inhibitors
6	Alpha‐adrenoreceptor antagonists
7	Digitalis glycosides
8	Vasodilators used in cardiac diseases
CNS‐active medications	
9	Antipsychotics
10	Anxiolytics
11	Hypnotics and sedatives
12	Antidepressants
13	Opioids
14	Antiepileptics
Others	
15	Anti‐Parkinson drugs—Anticholinergic agents
16	Anti‐Parkinson drugs—Dopaminergic agents
17	Drugs used in diabetes
18	Anti‐inflammatory and antirheumatic products, non‐steroids (NSAIDs)
19	Contact laxatives
	Bulk‐forming laxatives
	Osmotically acting laxatives
	Enemas
20	Proton pump inhibitors
